# Clinical and Radiological Characteristics of Non-Obese Female Patients with Idiopathic Intracranial Hypertension

**DOI:** 10.3390/jcm13061547

**Published:** 2024-03-08

**Authors:** Anat Horev, Gal Ben-Arie, Yair Zlotnik, Maor Koltochnik, Or Ben Chaim, Ron Biederko, Tamir Regev, Erez Tsumi, Ilan Shelef, Yana Mechnik Steen, Tal Eliav, Mark Katson, Erel Domany, Asaf Honig

**Affiliations:** 1 Department of Neurology, Soroka University Medical Center, Ben-Gurion University of the Negev, Beer Sheva 8410501, Israel; yairzl@clalit.org.il (Y.Z.); yaname@clalit.org.il (Y.M.S.); 2 Department of Diagnostic Imaging, Soroka University Medical Center, Ben-Gurion University of the Negev, Beer Sheva 8410501, Israel; galbe@clalit.org.il (G.B.-A.); ilans@clalit.org.il (I.S.); 3 Joyce and Irving Goldman Medical School, Faculty of Health Sciences, Ben-Gurion University of the Negev, Beer Sheva 8410501, Israelorbench@post.bgu.ac.il (O.B.C.); biederko@post.bgu.ac.il (R.B.); 4 Department of Ophthalmology, Soroka University Medical Center, Ben-Gurion University of the Negev, Beer Sheva 8410501, Israel; tamirre@clalit.org.il (T.R.); erezts@clalit.org.il (E.T.); 5 Faculty of Health Sciences, Ben-Gurion University of the Negev, Beer Sheva 8410501, Israel; talzus@post.bgu.ac.il; 6 Department of Neurology, Rambam Healthcare Campus, Haifa 3109601, Israel; m_katson@rmc.gov.il (M.K.); e_domany@rambam.health.gov.il (E.D.)

**Keywords:** idiopathic intracranial hypertension, obesity, severe papilledema, scleral flattening

## Abstract

While the typical patient with idiopathic intracranial hypertension (IIH) is an obese female of childbearing age, there are unique patient populations, such as non-obese females, that have not been well studied. Characterizing this subpopulation may increase awareness our of it, which may prevent underdiagnosis and improve our understanding of IIH’s underlying pathophysiology. We retrospectively reviewed electronic medical records and compared the clinical and radiological characteristics of non-obese (BMI < 30) and obese (BMI > 30) female patients with IIH. Two hundred and forty-six patients (age 32.3 ± 10) met our inclusion criteria. The non-obese patients (*n* = 59, 24%) were significantly younger than the obese patients (29.4 ± 9.9 vs. 33.2 ± 10.2, *p* = 0.004) and had higher rates of severe papilledema (Friesen 4–5; 25.4% vs. 11.8%, *p* = 0.019), scleral flattening (62.7% vs. 36.9%, *p* = 0.008), and optic nerve dural ectasia (78.0% vs. 55.6%, *p* = 0.044). Non-obese patients also had a tendency to have a higher lumbar puncture opening pressure (368 ± 92.7 vs. 344 ± 76.4, *p* = 0.062). Non-obese patients were three times more likely to present with a combination of scleral flattening and optic nerve dural ectasia (OR = 3.00, CI: 1.57–5.72, χ^2^ = 11.63, α < 0.001). Overall, non-obese females with IIH were found to have a more fulminant presentation, typified by higher rates of severe papilledema and radiological findings typical for IIH.

## 1. Introduction

Idiopathic intracranial hypertension (IIH) is defined as a lumbar puncture opening pressure ≥250 mmH_2_O with normal cerebrospinal fluid (CSF) findings and no evidence of hydrocephalus, mass lesions, or abnormal meningeal enhancement on MRI in the context of papilledema. Patients may also present without papilledema, given other radiographic findings, such as an empty sella and transverse venous sinus stenosis [1]. IIH can cause severe headaches and vision loss [2], and is typically found in obese women of a childbearing age [3,4,5,6]. Given the association with obesity, weight loss is one of the first treatment recommendations given to patients with IIH [7,8,9]. Recent studies describe unique subgroups among the IIH patient population: pediatric patients, adults above forty years old, men, and fulminant IIH patients. It is worth noting that these subgroups exhibit a significantly lower prevalence of obesity [10,11,12,13,14]. We assume that significant gaps remain in our understanding of these unconventional IIH groups. Furthermore, the causal role of obesity may vary between these groups. Given the notable presence of non-obese patients within reported IIH cohorts, we aimed to investigate and delineate this subgroup of patients, recognizing the potential value in understanding their unique characteristics.

## 2. Materials and Methods

### 2.1. Diagnosis of IIH

Patients were suspected of having IIH if they presented with clinical signs, including severe headache, tinnitus, elevated intracranial pressure (ICP), and a deterioration of vision. All patients were examined by a neurologist as well as a neuro-ophthalmologist, who performed a fundoscopy to look for signs of IIH (i.e., optic disc edema, 4th and 6th cranial nerve palsy). The severity of the papilledema was graded by neuro-ophthalmologists using the Friesen scale [15]. All cases with at least one eye showing severe papilledema (Friesen 4–5) were examined during admission by a neuro-ophthalmologist.

If a patient presented with increased ICP, computed tomography venography (CTV) or magnetic resonance venography (MRV) was used to rule out secondary causes, such as venous thrombosis. Imaging was reviewed by a neuroradiologist and an endovascular interventional neurologist within a day to evaluate the degree of venous sinus stenosis.

Following clinical and radiological evaluation, a lumbar puncture was performed on all patients suspected of having IIH. Rather than being placed in a lateral recumbent position, patients straightened their legs for the procedure. Their CSF cell count was analyzed to exclude further causes of elevated ICP. All patients were treated conservatively, according to the guidelines.

### 2.2. Study Population

We retrospectively reviewed all the medical charts of patients diagnosed with IIH at our institution. An electronic search of benign intracranial hypertension (ICD-9 code 348.2 corresponding to IIH) diagnoses between 2012 and 2022 was performed. We excluded males, patients <18 years old, patients with an LP opening pressure below 250 mmH2O, patients without available cerebral venous imaging (either CT or MRI venography) that was performed at the time of diagnosis, and patients who did not meet the Friedman’s criteria for IIH [16]. These exclusions were chosen to avoid the effect of other subpopulations (males, pediatrics, and those not included within Friedman’s revised criteria) on the study’s cohort.

### 2.3. Data Collection

Height, weight, and calculated BMI, as well as medical history, any recorded headache, the degree of papilledema, and LP opening pressure, were collected from the patients’ electronic medical records at the time of diagnosis. The variable “socioeconomic status” was determined by the Socioeconomic Index (SEI, which evaluates municipalities based on 14 factors such as income, education, and demographics. Municipalities are grouped into SEI clusters from 1 to 10, with lower scores indicating a lower socioeconomic status. The scores are automatically calculated in the hospital’s computerized medical system.

The collection of each patient’s medical history included a multitude of variables, such as Behçet’s disease, systemic lupus erythematosus (SLE), obstructive sleep apnea, Addison’s disease, and renal failure, among others. When presenting the data, we only referenced background diseases if we identified at least one patient with a diagnosis. For example, there were no patients with obstructive sleep apnea, so it has not been included in our tables. Similarly, we recorded all the medications patients were taking five years prior to their IIH diagnosis. While there are several medications relevant to IIH, such as tetracycline, demeclocycline, and acitretin, we presented only the medications used by our patients.

Radiological data were collected by a single radiologist, who reviewed the patients’ brain CTV/MRV performed at the time of diagnosis while blinded to the patients’ clinical data. The imaging was graded according to the following criteria: empty sella turcica (yes/no), scleral flattening (yes/no), optic nerve ectasia above 5 mm (yes/no), width measurement of both optic nerves, and the degree of bilateral transverse sigmoid junction venous stenosis (TSS), which was ranked using the combined conduit Farb score (CCS) [17].

The grading of patency was performed in each of the transverse sinuses (left and right) and ranked on a scale of 0 to 4, based on the degree of patency: 0 indicates 0% patency, 1 indicates less than 25% patency, 2 indicates 25–50% patency, 3 indicates 50–75% patency, and 4 indicates 75–100% patency. The scores for each side are then combined to calculate the CCS (ranging from 0 to 8). A healthy, normal result is 8, indicating either no or very minimal stenosis. A score below 5 is considered indicative of significant stenosis.

### 2.4. Statistical Analysis

Patients with obesity (BMI > 30) were compared to patients without obesity (BMI < 30). In the univariate analysis we used a Mann–Whitney test to compare socioeconomic status, the number of births, CSF opening pressures, and the FARB scores between the two groups, as well as the radiological findings and radiological signs of IIH (including the presence of scleral flattening, optic nerve sheath enlargement, and empty sella). Clinical features of IIH were compared between the study groups using a Pearson chi-square test. A logistic regression analysis was used to test for an association between the patient’s obesity as a binary variable (obese vs. non-obese) and all patient characteristics that were statistically significant in the univariate analysis (entry criteria *p* < 0.1 on univariate analysis). The result of the logistic regression was presented with the odds ratio (OR) and a CI of 95%.

## 3. Results

Two hundred and forty-six female patients (age 32.3 ± 10) met the inclusion criteria for the study (Table 1). Among the study population, 23.9% (*n* = 59) had a BMI < 30 (considered non-obese) and 32% (*n* = 19) had a BMI < 25 (considered normal weight), which accounted for 7.7% of the total study population. Upon diagnosis, non-obese patients (*n* = 59, 24%) were younger (29.4 ± 9.9 vs. 33.2 ± 10.2, *p* = 0.004) and had had a lower number of births (1.9 ± 2.2 vs. 3.0 ± 2.8, *p* = 0.015). The remainder of the baseline characteristics were similar between the two groups.

A comparison between the two groups upon diagnosis (Table 2) showed that non-obese patients tended to have a higher CSF opening pressure (368 ± 92.7 vs. 344 ± 76.4, *p* = 0.062) and had higher rates of severe papilledema (25.4% vs. 11.8%, *p* = 0.019) (Table 2). Recurrent hospitalizations with headache or IIH-related symptoms tended to be more common among non-obese patients (39.0% 26.2%, *p*-value = 0.071).

The neuroimaging features of the non-obese patients included higher rates of scleral flattening (62.7% vs. 36.9%, *p*-value = 0.008) and optic nerve dural ectasia (78.0% vs. 55.6%, *p* = 0.044) (Table 3). The combined FARB scores revealed a severe degree of cerebral sinus stenosis in both groups (2.5 ± 1.9 in the non-obese group and 2.6 ± 2.1 in the obese group, *p* = 0.93). Furthermore, when categorizing the binary combined FARB score into high-grade stenosis (1–2) and low-grade stenosis (3–8), no difference was found.

Upon multivariate analysis, scleral flattening was the only variable independently associated with non-obesity (OR 2.4, 95% CI 1.02–5.9. *p* = 0.004) (Table 4).

### Post Hoc Analysis

Because we found the presence of either scleral flattening or optic nerve dural ectasia to be strongly associated with the non-obese group, we examined their combined presence in each group. We found that non-obese patients were three times more likely to present with a combination of scleral flattening and optic nerve dural ectasia in comparison to obese patients (OR = 3.00, CI: 1.57–5.72, χ^2^ = 11.63, α < 0.001).

## 4. Discussion

In this study, we compared the clinical, radiological, and demographic features of non-obese and obese female patients with IIH.

Within our cohort, 23% of IIH patients were non-obese (BMI < 30) and 7.7% were of normal weight (BMI < 25). A previous large cohort of 1339 IIH patients presented similar rates: 26.1% were non-obese, and 9.8% had a normal BMI [5]. Several studies also describe similar rates of non-obese IIH patients; however, this specific subpopulation was not the main focus of these studies [18,19,20]. Moreover, men with IIH were found to have a higher rate of non-obesity than women [19].

Upon comparing their demographic features, we observed that IIH in non-obese patients was diagnosed at a significantly younger age: 25.8 (18.1; 64.5) versus 32.1 (18.3; 87.9) in the obese group (*p* = 0.004). Furthermore, when we focused on the group with normal BMIs (*n* = 19), the median age was lower than the non-obese age of diagnosis: 22.6 (18.6; 64.5). It is noteworthy that no other significant differences were identified across the demographic or medical history of the groups.

In our study, transverse-sigmoid stenosis was found to be severe in both groups. As in many other studies, these results highlight the importance of venous stenosis in the patho-mechanism of IIH [17,21,22]. In some studies, obesity is mentioned as a possible trigger or ameliorating factor for venous stenosis [21,23,24]. However, we found the non-obese population to have a very high degree of venous stenosis, suggesting that venous stenosis may not necessarily be caused or influenced by obesity.

We found that non-obese IIH patients, upon diagnosis, tended to present with a higher CSF opening pressure and higher rates of severe papilledema. These findings may suggest a more fulminant nature of the disease in non-obese patients. This observation has been reported in fulminant IIH patients in the past [14]. Moreover, non-obese neuroimaging features included higher rates of scleral flattening and optic nerve dural ectasia.

Furthermore, scleral flattening was independently associated with non-obesity in the multivariant analysis. These findings coincide with the higher LP opening pressure and higher degree of papilledema in this group, further supporting a more fulminant course of the disease. One possible explanation for a fulminant presentation in the non-obese group is a delay in diagnosis and treatment. Treating physicians might perceive these patients as atypical for IIH and, therefore, diagnose them at a more advanced stage of the disease. It is also possible that non-obese IIH patients fail to adjust to their increased ICP. This is evidenced by higher opening pressures, and, consequently, more severe manifestations of IIH. In contrast, we assume that obese IIH patients may have confronted an increased ICP in their past and developed ways to compensate for it. Rapid weight gain, rather than gradual, may pose a greater accommodative challenge for cerebral structures.

It has been suggested that body fat distribution affects IIH in non-obese patients. Several studies have described the effect of truncal fat distribution in IIH patients [11,19,25]. In our study, body fat distribution was not measured. Body fat distribution or body proportions are not necessarily reflected in the BMI and may affect the patho-mechanism of disease.

In this study, we demonstrated that a significant part of the IIH population is not obese. Although IIH is frequently described as a condition affecting young, obese women, our study, alongside other research, demonstrates that approximately 20% of patients are not obese. Moreover, we illustrated that, apart from their younger age, the non-obese patient group exhibited similar medical histories, symptoms, and radiological findings compared to the obese group. Notably, we observed even more prominent typical radiological features, such as scleral flattening and optic nerve dilatation, in the non-obese group. Being aware of this subgroup of patients is critical, as the non-obese population may be underdiagnosed or misdiagnosed, leading to potential delays in the appropriate treatment.

For many years, IIH has been regarded as a condition with well-defined criteria and a clearly delineated patient population. In recent years, however, an increasing number of publications have emphasized the necessity of viewing IIH as being on a spectrum of several subpopulations [26]. Our data support existing studies that indicate a significant variability in the IIH patient population. Recognizing IIH as a spectrum with a diverse patient population may suggest the existence of multiple patho-mechanisms of this disease. Consequently, there is a need to review the current diagnostic and treatment criteria and approaches.

Our study has several limitations. Due to the retrospective nature of our study, the data may not have been fully recorded. Furthermore, our study focuses on the patients’ BMI upon presentation and does not consider alterations in weight or the rate of change in their weight that have preceded the IIH diagnosis. Additionally, our data do not include measurements of body fat distribution and weight changes prior to IIH diagnosis. Future research, particularly prospective studies focusing on body fat distribution, is essential to deepen our understanding and improve the treatment of the population with IIH.

## Figures and Tables

**Table 1:** Baseline characteristics of obese and non-obese female patients with idiopathic intracranial hypertension.

		Non-Obese BMI < 30 (*N* = 59)	Obese BMI > 30 (*N* = 187)	*p*-Value
Age at diagnosis, mean ± SD		29.4 ± 9.9	33.2 ± 10.2	0.004
Median (min; max)		25.8 (18.1; 64.5)	32.1 (18.3; 87.9)	
Smoking, yes, *n* (%)		19 (32.2)	42 (22.5)	0.11
Drug use, yes, *n* (%)		1 (1.7)	3 (1.6)	1
Socioeconomic Status, mean ± SD		3.1 ± 2.8	3.2 ± 2.6	0.725
Median (min; max)		3.0 (0; 10)	3.0 (0; 9)	
Family Status, *N* (%)	Married	34 (57.6)	113 (60.4)	0.107
	Single	20 (33.9)	31 (16.6)	
	Divorced	4 (6.8)	15 (8.0)	
	Widowed	0 (0.0)	2 (1.1)	
Number of births				0.015
Mean ± SD		1.9 ± 2.2	3.0 ± 2.8	
Median (min; max)		1.0 (0; 8)	2.0 (0; 14)	
Anemia, yes, *n* (%)		6 (10.2)	23 (12.3)	0.833
Hypoparathyroidism, yes, *n* (%)		1 (1.7)	0 (0.0)	0.542
Polycystic Ovary Syndrome, yes, *n* (%)		2 (3.4)	10 (5.3)	0.793
Renal Failure, yes, *n* (%)		0 (0.0)	4 (2.1)	0.588
Lithium, yes, *n* (%)		1 (1.7)	0 (0.0)	0.542
Doxycycline, yes, *n* (%)		18 (30.5)	50 (26.7)	0.691
Minocycline, yes, *n* (%)		2 (3.4)	4 (2.1)	0.953
Isotretinoin, yes, *n* (%)		2 (3.4)	6 (3.2)	1

**Table 2:** Presenting features of obese and non-obese female patients with idiopathic intracranial hypertension.

	Non-Obese BMI < 30 (*N* = 59)	Obese BMI > 30 (*N* = 187)	*p*-Value
Headaches at diagnosis, *n* (%)	55 (93.2)	165 (88.2)	0.653
CSF opening pressure at diagnosis, mmH_2_O			0.062
Mean ± SD	368 ± 92.7	344 ± 76.4	
Median (min; max)	350 (250; 570)	320 (250; 600)	
Papilledema at presentation, *n* (%)	48 (81.4)	145 (77.5)	0.868
Severe papilledema at presentation, *n* (%)	15 (25.4%)	22 (11.8%)	0.019
Friesen scale, Mean ± SD	2.05 ± 1.68	1.5 ± 1.3	0.06
Recurrent hospitalization, yes, *n* (%)	23 (39.0)	49 (26.2)	0.071

**Table 3:** Idiopathic intracranial hypertension neuroimaging features of obese and non-obese female patients with idiopathic intracranial hypertension.

	Non-Obese BMI < 30 (*N* = 59)	Obese BMI > 30 (*N* = 187)	*p*-Value
Empty sella, *n* (%)	36 (61.0)	109 (58.3)	0.546
Scleral flattening, *n* (%)	37 (62.7)	69 (36.9)	0.008
Optic nerve dural ectasia, yes, *n* (%)	46 (78.0)	104 (55.6)	0.044
Optic nerve width, right			0.786
Mean ± SD	6.4 ± 0.7	6.4 ± 0.8	
Median (min; max)	6.0 (5; 8)	6.0 (5; 9)	
Optic nerve width, left			0.569
Mean ± SD	6.2 ± 0.8	6.3 ± 0.9	
Median (min; max)	6.0 (5; 8)	6.0 (5; 8)	
Combined FARB score			0.929
Mean ± SD	2.5 ± 1.9	2.6 ± 2.1	
(min; max)	2.0 (0; 8)	2.0 (0; 8)	
High-grade combined FARB score (0–2), *n* (%)	32 (54.2%)	88 (47.1%)	0.885

**Table 4:** Multivariate analysis of the variables associated with obesity.

	OR	95% CI	*p*-Value
Age at diagnosis	1.05	1.0–1.1	0.07
Number of births	1.09	0.9–1.3	0.3
Severe papilledema	0.77	0.33–1.8	0.6
CSF opening pressure at presentation	1.00	0.9–1.0	0.2
Sclera flattening	0.42	0.1–0.9	0.004
Optic nerve dural ectasia	0.75	0.2–2.0	0.6

OR = odds ratio, CI = confidence interval.

## Data Availability

The data presented in this study are available on request from the corresponding author.
